# Epigenetic Insights and Potential Modifiers as Therapeutic Targets in *β*–Thalassemia

**DOI:** 10.3390/biom11050755

**Published:** 2021-05-18

**Authors:** Nur Atikah Zakaria, Md Asiful Islam, Wan Zaidah Abdullah, Rosnah Bahar, Abdul Aziz Mohamed Yusoff, Ridhwan Abdul Wahab, Shaharum Shamsuddin, Muhammad Farid Johan

**Affiliations:** 1 Department of Haematology, School of Medical Sciences, Universiti Sains Malaysia, Kubang Kerian 16150, Malaysia; natikahzk@gmail.com (N.A.Z.); wanxzaidah@gmail.com (W.Z.A.); rosnahkb@usm.my (R.B.); 2 Department of Neurosciences, School of Medical Sciences, University Sains Malaysia, Kubang Kerian 16150, Malaysia; drazizmy@usm.my; 3 Department of Biomedical Sciences, Kulliyyah of Allied Health Sciences, International Islamic University Malaysia, Kuantan 25200, Malaysia; ridhwan@iium.edu.my; 4 School of Health Sciences, University Sains Malaysia, Kubang Kerian 16150, Malaysia; shaharum1@usm.my; 5 Institute for Research in Molecular Medicine (INFORMM), University Sains Malaysia, Kubang Kerian 16150, Malaysia; 6 USM-RIKEN Interdisciplinary Collaboration for Advanced Sciences (URICAS), Universiti Sains Malaysia, Penang 11800, Malaysia

**Keywords:** thalassemia, *β*–thalassemia, epigenetics, DNA methylation, *IGSF4*, *LARP2*, *BCL11A*, *KLF1*, *HBS1L-MYB*, HBG-Xmn1

## Abstract

Thalassemia, an inherited quantitative globin disorder, consists of two types, α– and *β*–thalassemia. *β*–thalassemia is a heterogeneous disease that can be asymptomatic, mild, or even severe. Considerable research has focused on investigating its underlying etiology. These studies found that DNA hypomethylation in the β–globin gene cluster is significantly related to fetal hemoglobin (HbF) elevation. Histone modification reactivates γ-globin gene expression in adults and increases β–globin expression. Down-regulation of γ–globin suppressor genes, i.e., *BCL11A*, *KLF1*, *HBG-XMN1*, *HBS1L-MYB*, and *SOX6,* elevates the HbF level. *β*–thalassemia severity is predictable through *FLT1*, *ARG2*, *NOS2A*, and *MAP3K5* gene expression. *NOS2A* and *MAP3K5* may predict the *β*–thalassemia patient’s response to hydroxyurea, a HbF-inducing drug. The transcription factors NRF2 and *BACH1* work with antioxidant enzymes, i.e., *PRDX1*, *PRDX2*, *TRX1*, and *SOD1*, to protect erythrocytes from oxidative damage, thus increasing their lifespan. A single *β*–thalassemia-causing mutation can result in different phenotypes, and these are predictable by *IGSF4* and *LARP2* methylation as well as long non-coding RNA expression levels. Finally, the coinheritance of *β*–thalassemia with α–thalassemia ameliorates the *β*–thalassemia clinical presentation. In conclusion, the management of *β*–thalassemia is currently limited to genetic and epigenetic approaches, and numerous factors should be further explored in the future.

## 1. Introduction

**Figure 1:**
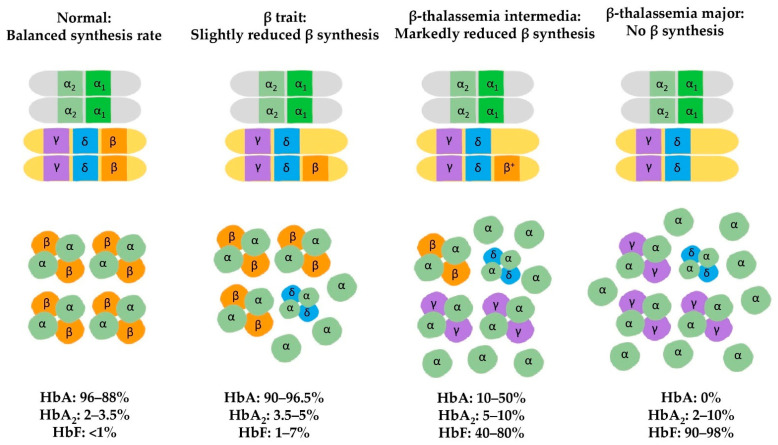
Imbalance of globin tetramers synthesis rate in *β*–thalassemia due to lack of subsequent genes (adapted from learnhaem.com accessed on 11 February 2021).

Thalassemia, a monogenic hematologic disease, is characterized by quantitative abnormalities in the globin chain of hemoglobin (Hb)—the oxygen-carrying component of red blood cells (RBCs). Adult HbA is a tetramer consisting of two alpha and two beta subunits (α_2_β_2_). Lacking these globin chains results in α– and *β*–thalassemia, respectively. In α–thalassemia, deletions of the α–globin gene are the leading cause of the disease, while point mutations in the β–globin gene mainly occur in *β*–thalassemia [1,2]. The deficiency of either α– or β–globin chains results in the inability of RBCs to form properly, so they cannot carry sufficient oxygen. In these patients, anemia begins in early childhood and lasts throughout life [3]. The globin chain imbalance in thalassemia is usually corrected by its globin tetrameric partner (Figure 1). The normal hemoglobin molecule mainly found in adults is HbA (α_2_β_2_), while HbA_2_ (α_2_δ_2_) can be found in small amounts in adults. In β–trait individuals, there is a slight reduction in β–globin synthesis, which causes an excess of α–globin chains due to the lack of β–globin binding partners. These excess α–globin chains are unstable, and precipitate in RBCs, leading to hemolysis. In response to the excess α–globin chains, the δ–globin gene is activated and produces δ–globin chains. The α–globin chains pair with the δ–globin chains, forming HbA_2_ (α_2_δ_2_). In *β*–thalassemia intermedia, β–globin synthesis decreases significantly. When there are insufficient δ–globin chains to pair with the excess α–globin chains, the γ–globin gene is activated and produces γ–globin chains, which form fetal hemoglobin (HbF; α_2_γ_2_). Thus, the level of HbF increases in adults with *β*–thalassemia intermedia. Finally, when there is no β–globin synthesis due to the lack of a functional β–globin gene, such as in *β*–thalassemia major, the RBCs mainly contain HbF.

Inheriting either the *β*–thalassemia mutation that encodes for highly unstable hemoglobin variants, or the mutation that encodes for extra copies of the α–globin gene, results in a lack of stable globin tetramers. Thus, the patient’s clinical condition deteriorates. In contrast, the co-existence of α– and *β*–thalassemia, and the increased production of γ–globin, generates additional fetal Hb (HbF), resulting in a less severe *β*–thalassemia presentation [4].

Hemoglobinopathies are genetic disorders in which point mutations produce structurally abnormal globin chains, such as Hb-S, -C, -D, and -E. In southeast Asia, HbE is the most commonly observed variant [5]. Concurrent inheritance of *β*–thalassemia and the HbE hemoglobin variant results in HbE/*β*–thalassemia, the hemoglobinopathy most common in many Asian countries and responsible for at least half of all severe *β*–thalassemia cases worldwide [5].

*β*–thalassemia is a complex disease in which the genotype does not necessarily define the disease outcome or the phenotype. The clinical and hematological spectrum of *β*–thalassemia, including *β*–thalassemia major and *β*–thalassemia intermedia, ranges from mild to severe [6]. Patients with a genotype usually associated with a mild form of the disease could present asymptomatic, mild, or severe symptoms. Those with a genotype typically associated with severe disease could present as mild or asymptomatic. Information about the underlying molecular basis of thalassemia can help explain this complexity. Epigenetic factors can also influence thalassemia severity, leading to differing presentations despite similar underlying mutations [7].

Epigenetics is the alteration of gene activity without a change in the DNA sequence, including DNA methylation and histone modifications. In mammalian DNA, cytosine residue methylation often occurs within clusters of CpG dinucleotides (CpG islands). CpG methylation silences genes by inhibiting the binding of transcription factors. In contrast, CpGs in actively transcribed gene promoter regions are predominantly unmethylated [8]. In histone modification, active gene transcription occurs when lysine residues in N-terminal tails become acetylated. Meanwhile, methylated lysine residues can be associated with either active or repressed genes [8].

Various epigenetic modifiers are also involved in the clinical presentation of *β*–thalassemia. Since *β*–thalassemia patients lack β–globin chains, one of the most frequent mechanisms for restoring optimal oxygen-carrying status is to induce γ–globin expression to regain the generation of HbF tetramers (α2γ2) [9]. This review focuses on the association with modifiers of β– and HbE/*β*–thalassemia, which either ameliorate or aggravate the clinical disease symptoms and long-term consequences.

## 2. Epigenetics and *β*–thalassemia

### 2.1. DNA Methylation

**Figure 2:**
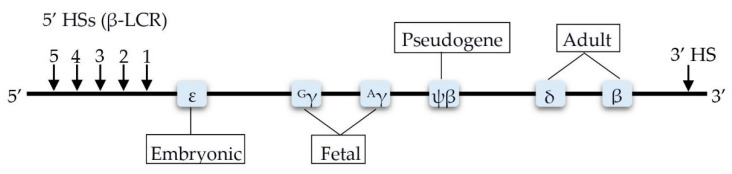
Schematic of β–globin cluster. HS, hypersensitive sites; LCR, locus control region.

The human β–globin cluster on chromosome 11 spans up to 70 kb and is composed of five genes (5′-*HbE*-*HBG2*-*HBG1*-*HBD*-*HBB*-3′; 5′-ε-γG-γA-δ-β–3′) and a distal regulatory element known as the locus control region (LCR) (Figure 2) [10].

Many have reported that fetal-to-adult Hb switching is controlled through the activity of epigenetic modulators such as *BCL11A*, *KLF1*, and *HBS1L-MYB* [11,12,13]. DNA hypomethylation and histone acetylation are effective in inducing γ–globin expression [14]. Bao et al. [15] described the hypomethylation patterns in the CpG sites around the LCR HS4-HS3 regions of the β–globin cluster as well as the γ– and β–globin promoters in β^0^/β^0^–thalassemia patients, compared with healthy controls. Hypomethylation was associated with high levels of HbF. Thus, this finding has increased our understanding of the association between methylation patterns and γ–globin expression in β^0^ thalassemia patients.

### 2.2. Histone Modification

Fard et al. [16] studied the effect of H3K27 methylation in erythroid progenitors derived from CD133^+^ cord blood stem cells. The cells were treated with the HbF-inducing drugs thalidomide and sodium butyrate, alone and in combination. Cells treated with thalidomide alone produced more hematopoietic colonies. They also had the greatest observed decrease in H3K27 methylation, 0.71-fold, compared to the negative control, dimethyl sulfoxide (*p* < 0.05). This result suggests that thalidomide has greater potential as a hypomethylating agent in treating sickle cell disease (SCD) and *β*–thalassemia. Vorinostat, a histone deacetylase (HDAC) inhibitor drug, was found to induce γ–globin production simultaneously with the suppression of α–globin. Nanomolar concentrations were sufficient to produce these synergistic effects without disturbing erythroid expansion, viability, differentiation, or the transcriptome, suggesting its potential as a *β*–thalassemia treatment [17]. Additionally, MS-275 is an HDAC inhibitor that induces HbF production. MS-275, in comparison to its analogs, was studied in the human K562 erythroleukemia cell line. Five novel agents (MD-20, -48, -61, -62, and -68) had a higher Hb-inducing activity compared to MS-275, and MD-48 had the highest Hb-inducing activity as it increased Hb up to 13.76-fold (*p* < 0.01) [18].

Another study found that the level of a novel candidate globin gene regulator, the histone methyltransferase protein *ASH1L,* correlated with the differentiation of human erythroid progenitor cells and β–globin gene expression. Peak *ASH1L* expression was reached on day six of erythroid differentiation, with increased *ASH1L* binding to promoter regions of the β–globin and α–globin genes. This result suggests that *ASH1L* may be a β–globin gene regulator and could modify *β*–thalassemia severity [19]. IOX1, a histone demethylase inhibitor, down-regulated α and α–like globin expression in a dose-dependent manner without influencing β–like globin expression, suggesting that IOX1 acts selectively on the α–globin locus. It is important to note that IOX1 is a pharmacologically feasible molecule, as it selectively suppresses α–globin expression without affecting erythroid differentiation and β–like globin expression. These findings suggest that IOX1 may be a lead compound for developing a *β*–thalassemia therapy by targeting this novel pathway [20]. In primary human erythroid and K562 cells, knockdown of the protein deacetylase *SIRT1* decreased γ–globin gene expression, while overexpression increased γ–globin expression. Since high levels of HbF reduce the severity of *β*–thalassemia, these findings show that SIRT1 may be a novel inducer of γ–globin expression for the treatment of severe *β*–thalassemia. *SIRT1* activator molecules, namely SRT2104 and SRT1720, have been shown to enhance and reactivate the γ–globin gene in cord blood and adult human erythroblasts, respectively. These small molecules have great potential as new HbF inducers. Dai et al. [21] suggested that *SIRT1* forms LCR loops contacting the γ–globin gene promoter, thus activating the gene. At the same time, the expression of γ–globin gene suppressors, such as *BCL11A*, *KLF1*, *HDAC1,* and *HDAC2,* stop, increasing histone acetylation on the γ–globin gene promoter.

## 3. Other Epigenetic Modifiers

### 3.1. IGSF4

*IGSF4*, located at 11q23.3, is a thalassemia-related gene that encodes cell adhesion molecules as a member of immunoglobulin superfamily 4. The gene has an important role in globin synthesis through its involvement in globin expression regulation [22]. A study by Yassim et al. [23] showed that 75% of *β*–thalassemia major patients (n = 21) and 95% of HbE/*β*–thalassemia patients (n = 12) had fully or partially methylated *lGSF4* promoter regions, respectively. Interestingly, the methylation status of the gene in HbE/*β*–thalassemia was significantly different (*p* < 0.05) from that of *β*–thalassemia major patients, which might contribute to the vast clinical variation in HbE/*β*–thalassemia patients [23,24]. In a case-control study in the Chinese population, methylation of the *IGSF4* gene promoter (containing 12 CpG sites) of thalassemia patients (n = 23) was significantly higher (*p* < 0.05) than that in healthy controls (n = 5). Confirmation by real-time PCR revealed that *IGSF4* expression was markedly down-regulated in the peripheral blood of thalassemia patients compared to that of the umbilical cord (ratio = 0.18) and peripheral blood (ratio < 0.50) of healthy controls. Thus, this study indicates that *IGSF4* could be a molecular biomarker for non-invasive prenatal diagnosis and clinical classification of thalassemia [22].

### 3.2. LARP2

The La ribonucleoprotein domain family (*LARP2*) gene, located at chromosome 4q28.2, is a ribonucleoprotein complex that acts mainly as an RNA binding protein, enabling the correct processing and maturation of RNA polymerase III transcripts as well as stimulating the initiation of translation. *LARP2* has a protective effect on HDACs. *LARP2* hypermethylation reduces or eliminates HDAC protection, which down-regulates or decreases the production of β–globin [25]. Additionally, in the promoter region of *LARP2*, Yassim et al. [24] detected partial methylation in HbE/*β*–thalassemia (43%) and *β*–thalassemia major patients (17%), where the 5′ cytosine of CpG 3 and CpG 5 were mostly partially methylated out of 18 CpGs. This partial methylation may contribute to the clinical variability of HbE/*β*–thalassemia presentation. In 75% of HbE/*β*–thalassemia patients (CD26/IVS1-5), decreased expression of *LARP2* was observed. Meanwhile, the gene was up-regulated in 80% of *β*–thalassemia major patients (IVS1-5/IVS1-5), where both groups were partially methylated and unmethylated, respectively. *LARP2* expression (1.49 ± 26.60) in HbE/*β*–thalassemia was not significantly different from normal controls or *β*–thalassemia major patients (*p* > 0.05). However, *LARP2* expression was significantly up-regulated in *β*–thalassemia major patients compared to normal controls (6.80 ± 16.42, *p* < 0.05). This finding suggests that HbE contributes to different pathophysiology in different types of *β*–thalassemia [26]. Finally, in a Chinese cohort, the *LARP2* promoter region was significantly hypermethylated (*p* < 0.05) in *β*–thalassemia (n = 10) when compared to normal individuals (n = 3), and gene expression of *LARP2* was also significantly decreased in *β*–thalassemia patients (*p* < 0.05) [25].

## 4. Factors Involved in the Transcription Control of the HBB Locus

**Figure 3:**
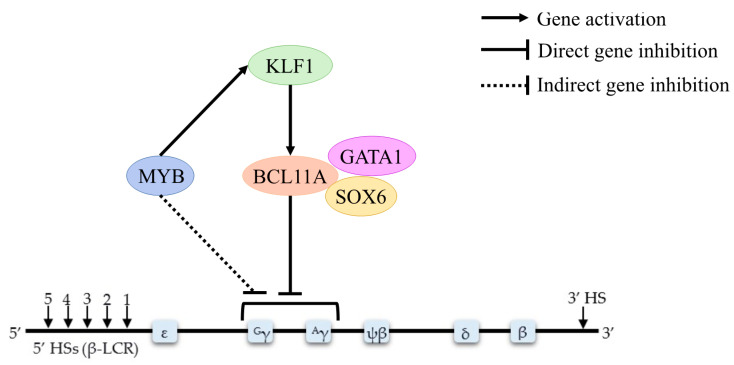
Regulators of the γ–globin gene, potentially inducing HbF production. *BCL11A* acts as a γ–globin silencer, and it is positively regulated by *KLF1*. MYB indirectly modulates HbF expression through the alteration of erythroid differentiation kinetics, and MYB also directly activates *KLF1* and other repressors.

The transcriptional switch from embryonic, to fetal, and then to adult globin gene expression requires elegant interactions between transcription factors such as *BCL11A*, KLF1, MYB, and *GATA1* (Figure 3).

### 4.1. BCL11A

The B-cell lymphoma 11 A (*BCL11A*) gene, located at 2p16.1, encodes a zinc-finger protein that serves as a major regulator of Hb switching and HbF silencing [27]. Down-regulation of *BCL11A* in primary human erythroid cells leads to robust HbF expression [11]. Mice lacking *BCL11A* fail to silence HbF expression in adult erythroid cells [28]. Additionally, robust HbF induction through the inactivation of *BCL11A* in humanized SCD mice corrects SCD-associated hematologic and pathologic defects [29]. These studies genetically and functionally validated *BCL11A* as a transcriptional regulator of HbF switching and silencing. To alleviate anemia in *β*–thalassemia major patients, Li et al. [27] reactivated HbF expression via lentiviral RNA interference, (RNAi)-mediated down-regulation of *BCL11A* in the K562 cell line, and an in vitro culture of erythroblasts derived from the mononuclear cells of normal individuals (n = 3) and *β*–thalassemia major patients (n = 3). Their findings showed that the down-regulation of *BCL11A* increased HbF levels in K562 cells and human erythroid cells from normal controls and *β*–thalassemia major patients, without any impact on erythroid maturation. Together, these findings emphasize the role of *BCL11A* as a quantitative regulator of HbF silencing.

Furthermore, Roosjen et al. [30] compared single and double knockdowns of *BCL11A* and DNA methyltransferase 1 (*DNMT1*), mediated by RNAi in the murine erythroleukemic (MEL) cell line. Compared with single knockdowns, double knockdowns of *BCL11A* and *DNMT1* significantly increased γ–globin expression to up to 90% of total β–like globin species (*p* < 0.001), suggesting that both *BCL11A* and *DNMT* act synergistically to elevate fetal globin expression in adult erythroid cells [30]. In a study of the fetal-to-adult Hb switch, erythroblasts from bone marrow significantly expressed *BCL11A* compared to fetal erythroblasts. Increased *HBG2* promoter methylation led to a decrease in the *HBG2*/*HBB* gene expression ratio. Additionally, single nucleotide polymorphisms (SNPs) at the β–globin locus, especially at the *BCL11A* binding site, and DNA methylation have independent effects on fetal hemoglobin expression in adult erythroblasts. For example, an analysis of the association between HbF levels and genotype revealed a strong deviation from the null distribution for *BCL11A* variants. A similar deviation pattern also appeared when only SNPs overlapping erythroid enhancer genes were analyzed [31]. In an Indonesian cohort, the minor allele of *BCL11A* SNP rs11886868 was associated with increased HbF concentration in HbE/*β*–thalassemia patients (n = 118), while the minor allele of rs766432 was associated with an even greater increase in HbF (4.7% more) [32]. In contrast, in a Thai cohort, there was no association between three *BCL11A* SNPs (rs1427407, rs10189857, and rs11886868) and HbF levels in HbE/*β*–thalassemia (n = 45) and homozygous HbE (n = 50) patients [33]. In severe (n = 383) and mild (n = 235) HbE/β^0^-thalassemia in Thai and Thai–Chinese patients, rs766432 was significantly associated with disease severity (*p* = 5.87 × 10^−10^) and HbF level (*p* = 1.00 × 10^−7^). However, SNPs in *BCL11A* explained only 3.3% of the variance in HbF levels, suggesting that other factors also impact HbF levels in HbE/β^0^-thalassemia patients [34].

Microarray analysis of CD34^+^-derived erythroid cells from the hereditary persistence of fetal hemoglobin deletion type-2 (HPFH-2) patients (n = 2), δ*β*–thalassemia patients (n = 2), and healthy controls (n = 3), demonstrated up-regulation of 12 microRNAs (miRNAs) targeting *BCL11A*, namely, *miR-21*, *miR-23b*, *miR-29a*, *miR-29b*, *miR-29c*, *miR-146a*, *miR-146b-5p*, *miR-148a*, *miR-148b*, *miR-128*, *miR-181a*, and *miR-590-5p*. These findings suggest that *BCL11A* gene expression is down-regulated in HPFH-2 compared to δ*β*–thalassemia and indicate a possible mechanism underlying these observations [35]. Mahdavi et al. [36] investigated the in vitro response of *BCL11A* to HU treatment in hematopoietic erythroid progenitors derived from the peripheral blood of homozygous IVSII-1G>A *β*–thalassemia patients (HU responders, n = 10, and HU non-responders, n = 10) and healthy individuals (n = 10). All groups showed a significant decrease in *BCL11A* expression post-treatment (HU responders: *p* < 0.001, HU non-responders: *p* = 0.048, controls: *p* < 0.001), also demonstrated by the elevation of total hemoglobin levels in all groups. However, the prediction of HU response by an in vitro method is not recommended since no difference in HU response was observed between the responders and non-responders (*p* = 0.334). Finally, the Chinese naturopathic medicine, yisui shengxue, down-regulated *BCL11A* expression in *β*–thalassemia patients (n = 8), with pre-treatment *BCL11A* expression significantly higher than post-treatment (*p* < 0.05). This suggests that yisui shengxue granules may have clinical efficacy for *β*–thalassemia patients by reducing *BCL11A* expression, which in turn increases the level of HbF and ameliorates the clinical symptoms of *β*–thalassemia [37].

### 4.2. HBS1L-MYB

*HBS1L-MYB* falls in a 126-kb intergenic region at chromosome 6q and contains erythroid trait-associated SNPs. The function of *HBS1L* in RBC development is uncharacterized; meanwhile, the *MYB* gene encodes a transcription factor that is essential for hematopoiesis and erythroid differentiation. MYB plays an essential role in controlling the erythroid cellular proliferation/differentiation balance, and regulates HbF levels through an undefined mechanism [12]. Nevertheless, two possible mechanisms for HbF expression via MYB modulation have been proposed: (1) indirect influence through alteration erythroid differentiation kinetics, by which low *MYB* levels accelerate erythroid differentiation, leading to release of early erythroid progenitor cells that synthesize HbF predominantly; and (2) direct influence via the activation of *KLF1* and other repressors (e.g., the nuclear receptors TR2/TR4) of γ–globin genes [38].

The RNAi-mediated double knockdown of *MYB* and *DNMT1* in an in vitro model of the MEL cell line resulted in a significant induction of ε–globin (*p* < 0.001), making up as much as 20% of the total β–like globin species compared to knockdowns of either *MYB* and *DNMT1*, suggesting that *MYB* and *DNMT1* cooperate to developmentally suppress embryonic β–like globin genes in adults [30]. In an Indonesian cohort, rs9399137 in *HBS1L-MYB* in *β*–thalassemia (n = 71) and HbE/*β*–thalassemia patients (n = 118) was not associated with either the HbF level or clinical appearance [32]. In a Thai cohort, however, significant differences in the allele frequencies of the *HBS1L-MYB* SNPs rs4895441 (*p* = 0.041) and rs9399137 (*p* = 0.048) were detected in homozygous HbE subjects (n = 50) with low (≤5%) and high (>5%) HbF levels. In addition, in homozygous HbE subjects, rs9399137 was significantly associated with differences in mean corpuscular volume (MCV; *p* = 0.005) and trended toward significant associations with mean corpuscular hemoglobin (MCH) (*p* = 0.057) and HbF levels (*p* = 0.051). These results suggest that rs9399137 plays a role in the variation of HbF production and hematological parameters, specifically in homozygous HbE subjects, but not in HbE/*β*–thalassemia patients [33]. Another *HBS1L-MYB* SNP, rs9376092, was significantly associated with disease severity (*p* = 2.36 × 10^−10^) in severe (n = 383) and mild (n = 235) HbE/β^0^-thalassemia cases in Thai and Thai–Chinese patients. rs9376092 is in strong linkage disequilibrium with rs4895441, which was significantly associated with HbF levels, suggesting that rs9376092 also plays a role in predicting HbE/β^0^-thalassemia severity. Additionally, variance component analysis revealed that *HBS1L-MYB* SNPs explained 10.5% of HbF levels, indicating that other factors also influence them [34].

### 4.3. KLF1

*KLF1* is a transcription factor essential for facilitating erythropoiesis and regulating the expression of several erythroid-specific genes. *KLF1* is a zinc-finger DNA binding protein that binds specifically to the 5′-CCMCRCCCN-3′ motif on most erythroid-specific genes. Furthermore, *KLF1* acts as a positive regulator of *BCL11A* transcription factor expression to control the levels of HbF [39].

An early study of *KLF1* revealed a direct influence on *BCL11A* levels and γ–globin/β–globin expression ratios, by directly activating β–globin and indirectly repressing γ–globin expression. This study suggested that the controlled knockdown of *KLF1* in adult erythroid progenitor cells may activate HbF expression in individuals with *β*–thalassemia or SCD, ameliorating their symptoms [13]. Wang et al. [40] provided further evidence that *KLF1* plays a significant role in controlling HbF levels, in a study involving 140 Chinese samples with high HbF levels (>1.5%), with the cohort consisting of patients with coexisting α–thalassemia (n = 12), *β*–thalassemia (n = 55), and 73 samples with no evidence of hemoglobinopathies other than elevated HbF. Five of these samples showed mutations in the *KLF1* gene, but the HbF level varied from 1.9% to 11.4% in subjects bearing the same *KLF1* mutation. However, there were no *KLF1* mutations in a matched control group of 110 samples referred for thalassemia screening but with normal HbF levels (<1.0%). These findings show that *KLF1* mutations elevate HbF levels. In another cohort of patients with hemoglobinopathies (n = 131) and elevated HbF levels, 11 different mutations in the *KLF1* gene were observed. Functional *KLF1* mutations were not identified in a matched cohort of 121 samples with normal HbF levels. Eight of the *KLF1* mutations were missense variants, one in exon 1 (L51R) and seven in the zinc-finger domains (R301C, R301H, W313C, R328H, R328L, T334K, and T334R), which are considered to disrupt DNA binding. Two were frameshift mutations in exon 2: an 11-bp deletion (K54PfsX9) and a 7-bp insertion (G176AfsX179), both producing downstream stop codons. The latter mutation occurred in two patients, both on its own and as a compound heterozygote with the L51R missense mutation. The final mutation identified in this group was a 1-bp nucleotide substitution (c.913+1G > A) at the 3′ end of exon 2, which is predicted to disrupt splicing [41]. In another Chinese cohort, the prevalence of *KLF1* mutations was found to be significantly higher (1.25% vs. 0.08%) in patients from the endemic thalassemia region in south China (n = 3839) than in patients in the non-thalassemia endemic region in north China (n = 1190). There were seven *KLF1* functional mutations in southern China: four previously reported (p.Gly176AlafsX179, p.Ala298Pro, p.Thr334Arg, and c.913+1G > A) and three novel variants (p.His299Asp, p.Cys341Tyr, and p.Glu5Lys). The two most common mutations, accounting for 90.6% of the total, were p.Gly176AlafsX179 and p.His299Asp. Furthermore, Liu et al. [42] validated that most non-thalassemic individuals had borderline MCV and MCH values due to KLF1 mutations, leading to an increase in HbF production and an amelioration of the clinical severity of *β*–thalassemia.

The latest findings by Fanis et al. [39] revealed a novel missense mutation in the second zinc-finger domain of the KLF1 protein, in two siblings who were predicted to have *β*–thalassemia major based on their genotype (IVS1-110); however, they were both transfusion-free and healthy. Another two family members also exhibited a high level of HbF while carrying a similar *KLF1* mutation. The inheritance of this heterozygous *KLF1* variant (NM_006563.4:c.968C>T, NP_006554.1:p.Ser323Leu) might play a vital role in ameliorating a severe *β*–thalassemia genotype, especially in homozygous IVS1-110 *β*–thalassemia. However, additional studies are warranted to confirm this hypothesis. To correlate the *KLF1* genotype with the phenotypic appearance of *β*–thalassemia, Hariharan et al. [43] performed an investigation of 370 individuals with different hemoglobinopathy conditions, and found that 8.1% had *KLF1* mutations. These *KLF1* mutations also contributed to borderline HbA2 cases (7.6%). Additionally, in patients bearing *KLF1* gene mutations, reduced *KLF1* and *BCL11A* expression correlated with increased γ–globin gene expression, supporting the idea that *KLF1* plays a role in regulating γ–globin gene expression [43].

One reliable hematologic parameter in *β*–thalassemia carriers is elevated HbA2 (4.0–6.0%). However, in some cases, *β*–thalassemia carriers present with a borderline HbA2 level (3.1–3.9%) that is difficult to identify. The involvement of *KLF1* gene variants in borderline HbA2 in *β*–thalassemia carriers was examined in a Saudi cohort (n = 212) since they have a high borderline incidence of HbA2. Borgio et al. [44] revealed that a borderline HbA2 level is not specific to any one type of *β*–thalassemia or β^+^-thalassemia variation in Saudis. Four variants in *KLF1* were detected: two exonic (c.304T > C and c.544T > C) and two 3′ untranslated region (3′UTR) variants (c.*296G > A and c.*277C > G). However, none of these are significantly associated with HbA2 levels. On the other hand, they found that the α–globin genotype, –α_2_3.7/α_1_α_2_, was the most frequent (55.55%) among healthy Saudis with borderline HbA2 levels. Therefore, a thorough investigation is needed to find other factors contributing to the borderline HbA2 level in the remaining 41.67% of Saudi *β*–thalassemia carriers. The role of *KLF1* in primary human fetal erythroid cells, which express both γ– and β–globin mRNA, was elucidated by investigating CD34^+^ umbilical cord blood cells (UCB erythroblasts). *KLF1* binds to the β–globin LCR and promoter, while *KLF1* is only bound to a small degree at the γ–globin promoter. Lentiviral-mediated *KLF1* knockdown in UCB erythroblasts diminished the active histone mark H3K4 me3 and RNA polymerase II recruitment at the β–, but not at the γ–globin, gene, thus increasing the transcription of γ–globin mRNA. The amount of *KLF1* expression is also weakly positively correlated with *BCL11A* mRNA; thus, manipulating *KLF1* and/or *BCL11A* is a potential therapy for *β*–thalassemia [45]. Lentiviral-mediated *KLF1* knockdown of normal (n = 3) and HbE/β^0^-thalassemia (n = 3) erythroblasts revealed that the expression of the γ–globin gene suppressor *BCL11A* decreased due to the absence of *KLF1*, which in turn activates the γ–globin gene and elevates HbF levels. Surprisingly, even though HbE/β^0^-thalassemia patients with *KLF1* mutations had elevated HbF, all patients in this study exhibited severe phenotypes, suggesting that the ability of *KLF1* mutations to elevate HbF was not sufficient to ameliorate the clinical appearance of HbE/β^0^-thalassemia [46].

### 4.4. GATA1

*GATA1* is the main transcription factor in erythropoiesis. It is crucial for erythroid maturation at an early stage, and its down-regulation is necessary to terminate erythroid differentiation [47]. Lessard et al. [31] observed enrichment of *GATA1* binding sites in fetal erythroblasts, suggesting that *GATA1* preferentially binds to hypomethylated sites in fetal erythroblasts. In an in vivo footprint analysis of *GATA1*, no significant footprint was observed in adult erythroid cells or lymphocytes when the γA promoter was methylated. However, in the absence of methyl groups, strong *GATA1* binding site footprints were detected despite there being no CpG sites within *GATA1*, signifying that methylation does not directly influence protein-DNA binding but is rather mediated by histone deacetylation [48]. Furthermore, treatment of K562 cells with yisui shengxue granules decreased *GATA1* and *GATA2* expression, suggesting that yisui shengxue granules should be explored for therapeutic development [49].

### 4.5. FLT1

*FLT1* encodes the vascular endothelial growth factor receptor *FLT1*, which is a tyrosine kinase and regulates cell proliferation and differentiation. The *FLT1* variant rs2182008 (G>A) is associated with elevated HbF levels (*p* = 0.039) among non-transfusion-dependent (NTDT) Hellenic-origin *β*–thalassemia patients (n = 7). This suggests that rs2182008 is a potential biomarker for predicting *β*–thalassemia severity, since patients with the effect allele exhibit a mild phenotype and have a reduced dependency on blood transfusions [50].

### 4.6. BACH1

In a Malaysian cohort of 47 HbE/*β*–thalassemia patients, *BACH1* expression significantly correlated with age (*p* = 0.006) as well as α– (*p* = 0.002), β– (*p* = 0.001), and γ–globin (*p* = 0.001) gene expression levels and heme oxygenase-1 protein level (*p* = 0.001). This finding indicated that up-regulation of *BACH1* expression occurs due to a compensatory effect, to restore the globin chain equilibrium and minimize the effects of oxidative stress in HbE/*β*–thalassemia patients [51]. A summary of the epigenetic modifiers involved in *β*–thalassemia is presented in Table 1.

## 5. Genetic Findings

### 5.1. HBG2-Xmn1

The *Xmn1* (C > T) polymorphism in the promoter of the ^G^γ–globin gene, located on chromosome 11, is a *β*–thalassemia modifier associated with elevated HbF, consequently causing a milder phenotype of *β*–thalassemia. Additionally, carriers of the *Xmn1* (C > T) polymorphism positively react to HU drug treatment, to reduce their dependency on frequent blood transfusions [52].

In an Indonesian cohort, the *Xmn1* C allele demonstrated a significant modifying effect on HbF level and clinical presentation in HbE/*β*–thalassemia patients, but not in *β*–thalassemia patients [32]. In an Indian cohort, the *Xmn1* variant was significantly more common in patients with non-transfusion-dependent *β*–thalassemia (NTDT) (n = 139) than in cases with transfusion-dependent *β*–thalassemia (TDT) (n = 104; *p* < 0.001). Higher HbF levels were observed in those with the *Xmn1* polymorphism, suggesting that the *Xmn1* mutation improves HbE/*β*–thalassemia symptoms [53]. Association analysis of *Xmn1* with health-related quality of life (HRQoL) in TDT and HbE/*β*–thalassemia patients in a Malaysian cohort demonstrated that the *Xmn1* genetic modifier was significantly related to physical and emotional parameters (*p* = 0.008 and *p* = 0.013, respectively). Additionally, *Xmn1* was associated with physical and emotional function (*p* = 0.023 and *p* = 0.017, respectively), suggesting that effective HRQoL development strategies should be explored and implemented in healthcare centers, to increase the HRQoL of the patients without compromising treatment regimens [54]. Two groups of HbE/*β*–thalassemia patients in a Thai cohort with HbF levels <15% (n = 18) and ≥15% (n = 40) had a statistically significant difference in the frequency of the *Xmn1* polymorphism (*p* = 0.007). However, hematological aspects were not different between groups, strengthening the idea that *Xmn1* polymorphism plays a role in HbF production but not in anemia severity, as displayed by hematological parameters [55]. Additionally, a significant difference (*p* = 0.0001) in transfusion frequency was observed between subjects with or without (+/−) the *Xmn1* polymorphism in another Malaysian cohort of thalassemia major (n = 111), thalassemia intermedia (n = 21), HbE/*β*–thalassemia (n = 131), and HbE homozygous patients (n = 1). In that study, the *Xmn1* polymorphism (+/−) was more common in HbE/*β*–thalassemia patients, and patients without the *Xmn1* polymorphism (−/−) required more frequent transfusions each month than patients with other *Xmn1* genotypes (*p* = 0.022), demonstrating that this variant alleviated the clinical presentation and reduced the need for blood transfusions [52]. HbE/*β*–thalassemia responders (n = 78) to HU showed the strongest association (*p* < 0.0001) between the *Xmn1* polymorphism and an increase in HbF and Hb levels: a higher prevalence of *Xmn1* was observed among responders, including 23.08% (n = 18) who were homozygous and 66.67% (n = 52) who were heterozygous for the *Xmn1* polymorphism compared to 18% prevalence in the normal population (n = 100) and 15.62% in non-responders (n = 5). This suggests that *Xmn1* is a factor contributing to a good response to HU in elevating HbF levels, and thus ameliorating HbE/*β*–thalassemia presentation while reducing the need for regular blood transfusions [56].

### 5.2. α–Thalassemia Coinheritance

Coinheritance of α– and *β*–thalassemia could restore the balance of globin chains in *β*–thalassemia, where there is an inherent imbalance due to the lack of β–globin chains. Out of 87 *β*–thalassemia patients in a genotype–phenotype study in Malaysia, six coinherited α–thalassemia mutations, namely α^3.7^ and α^SEA^. HbE/*β*–thalassemia patients with α^SEA^ had a less frequent need for blood transfusions than HbE/*β*–thalassemia patients with α^3.7^ or without α–thalassemia coinheritance, demonstrating that α–thalassemia coinheritance had an ameliorating effect on the severity of HbE/*β*–thalassemia [52]. Among HbE/*β*–thalassemia patients (n = 82) in an Indian cohort, 19 patients had a deletion of one α–globin gene (-α/αα) (α^3.7^ or α^4.2^), and four patients lacked two α–globin genes (--/αα or -α/-α) (α^3.7^ and α^4.2^). These included nine excellent HU responders who demonstrated an increase in Hb level that was significantly correlated (*p* < 0.01) with a decrease in transfusion regularity. The decreases in transfusion frequency were 74.27% in good HU responders and 100% in excellent responders, compared to other good responders who had complete α–globin gene sets. In addition, moderate and poor HU responders had no significant reduction in transfusion frequency pre- and post-therapy (*p* < 0.05), and none had α–globin gene deletion. Therefore, an excellent response to HU therapy is expected based on the α–globin gene deletion status in thalassemic patients [57]. In another cohort of Indian HbE/*β*–thalassemia patients (n = 240) divided into three severity-based groups, α deletions or mutations were found in 12.9% (n = 31) of the recruited patients: 22 and nine patients were in the mild and moderate groups, respectively, meanwhile, α triplication was found in 4.6% (n = 11) of the HbE/*β*–thalassemia patients, including eight patients from the severe group and three patients from the moderate group. In terms of α–globin gene numbers, it is important to note that α deletions or mutations had ameliorating effects, while α triplication had deleterious effects on HbE/*β*–thalassemia patients, since patients have mild phenotypes if they inherit α deletions or mutations, while α triplication leads to severe HbE/*β*–thalassemia [58]. In a large cohort of Thai HbE/*β*–thalassemia patients (n = 925), coinheritance of deletional α–thalassemia (n = 81) was found to be responsible for a mild clinical phenotype with fewer or no blood transfusions, and a consistent finding of α triplication only in the severe group of HbE/*β*–thalassemia patients with splenectomy (n = 4), validating the fact that the clinical presentation of *β*–thalassemia becomes more severe with a greater ratio of globin chain tetramers [59].

### 5.3. ARG2

Arginase 2 (*ARG2*) protects erythroid cells from oxidative stress caused by nitric oxide. HU treatment ameliorates *β*–thalassemia through the synthesis of HbF, which increases the level of HbF markedly [60]. *ARG2* is associated with HU therapy response in *β*–thalassemia patients. In a comparison between healthy subjects (n = 53) and NTDT *β*–thalassemia patients (n = 7), *ARG2* variant rs10483801 was significantly associated with elevated HbF levels (*p* = 0.001), suggesting that *ARG2* may be a predictor for a mild *β*–thalassemia phenotype [50].

### 5.4. NOS2A

The nitric oxide synthase 2a (*NOS2A*) gene is also associated with HU therapy response through the activation of HbF. Nitric oxide synthases (NOS) utilize L-arginine and molecular oxygen to generate nitric oxide and L-citrulline. NOS and γ–globin levels are known to decrease during erythroid cell differentiation. A comparison between healthy individuals and *β*–thalassemia major patients revealed that *NOS2A* variant rs1137933 was associated with a severe phenotype (*p* = 0.008) in these patients [61].

In erythroid cell maturation, *NOS2A* gene expression decreases along with a decrease of γ–globin, reducing the formation of HbF. Upon HU therapy in HbS (HBB: c.20A>T)–*β*–thalassemia compound heterozygous patients (n = 42), the *NOS2A* rs944725 variant was associated with an increase in HbF (*p* = 0.015), indicating that *NOS2A* gene expression may be another marker for a positive response to HU therapy [50].

### 5.5. MAP3K5

*MAP3K5* is a member of the p38 and Jun N-terminal kinase (JNK) mitogen-activated protein kinase (MAPK) pathway and is important for cellular stress response. The p38 and JNK MAPK pathway has been proposed to be involved in the induction of HbF production mediated by HU therapy. As in HU therapy for Hellenic-origin HbS (HBB: c.20A>T)-*β*–thalassemia patients (n = 42), a strong association (*p* = 0.004) between a *MAP3K5* gene promoter short tandem repeat and an increase in HbF levels was observed as a response to HU therapy. The presence of an extra GCGCG repeat may decrease *MAP3K5* gene expression, possibly by disruption of the p38 and JNK MAPK pathway, consequently leading to the absence of response to HU treatment. This suggests that *MAP3K5* gene expression is an indicator of HU therapy response [50]. Furthermore, a short tandem repeat in the *MAP3K5* promoter and two intronic *MAP3K5* gene variants (rs9483947 and rs9376230) were analyzed in *β*–thalassemia major (n = 92), *β*–thalassemia minor (n = 11), and healthy controls (n = 94). These genetic variants are associated with low HbF levels and a severe disease phenotype. High HbF levels are associated with the up-regulation of *MAP3K5* expression, where HU can affect the *MAP3K5* expression. This supports the argument that *MAP3K5* polymorphisms allow a positive response to HU treatment in ameliorating the *β*–thalassemia phenotype [62].

## 6. Putative Targets of Therapeutic Interventions

### 6.1. NRF2

Since *β*–thalassemia is an iron-associated disease, therapeutic approaches targeting the iron homeostasis pathway have been explored. For instance, NRF2 is a transcription factor responsible for maintaining cellular health by providing intrinsic protective antioxidant properties. In a β–thalassemic mouse experiment by Lim et al. [63], NRF2 activation was caused by excessive iron, leading to hepcidin synthesis by hepatocytes through *Bmp6* expression. That study demonstrated that NRF2 mediated the connection between cellular health and systemic iron homeostasis and was responsible for reducing iron-induced oxidative damage. In a Thai cohort of pediatric HbE/*β*–thalassemia patients (n = 24), iron overload, elevated lipid peroxidation, and marked diminution in the reduced glutathione (GSH) level were observed. A key enzyme for GSH biosynthesis, glutamate-cysteine ligase catalytic (GCLC) subunit level, was elevated in HbE/*β*–thalassemia patients compared to healthy controls (n = 22). Additionally, GCLC elevation in plasma was correlated with serum iron levels, suggesting that the expression of GCLC is an adaptive response to oxidative stress caused by iron overload in HbE/*β*–thalassemia [64]. NRF2 interacts with KEAP1 to form the NRF2/ KEAP1 complex in response to oxidative stress during erythrocyte development; KEAP1 oxidation allows NRF2 release turn, up-regulates *PRDX1* and *SOD1* production. In a Brazilian cohort, increased levels of the NRF2/KEAP1 complex were observed in *β*–thalassemia intermedia (n = 15) patients where the expression of NRF2 was more prominent (approximately a 3-fold change) than KEAP1 (approximately a 2-fold change), supporting the critical role of NRF2 in compensating for oxidative stress [65].

### 6.2. PRDX1

Reactive oxygen species (ROS) generation in erythrocytes is one pathophysiology of *β*–thalassemia. The peroxiredoxin 1 (*PRDX1*) gene, located at 1p34.1, encodes antioxidant enzymes that reduce hydrogen peroxide, protecting red cells from oxidative damage. *PRDX1* gene expression and protein levels were found to be elevated in the reticulocytes and erythrocytes of *β*–thalassemia intermedia patients (n = 15) compared with healthy individuals (n = 16). In *β*–thalassemia major patients (n = 8), however, the up-regulation of *PRDX1* was limited to gene expression, suggesting that high ROS levels in *β*–thalassemia major patients degrade *PRDX1* transcripts, hindering protein production [65].

### 6.3. PRDX2

The peroxiredoxin 2 (*PRDX2*) gene, located at 19p13.13, is abundant in erythrocytes and protects RBCs from oxidative stress, especially during erythropoiesis [66,67]. *PRDX2* works together with *PRDX1* to reduce oxidative damage in the circulating erythrocytes of *β*–thalassemia intermedia patients by detoxifying ROS. In a cohort study by Romanello et al. [65], the transcript levels of *PRDX2* were reduced in the reticulocytes of *β*–thalassemia intermedia patients (n = 15), implying that peak *PRDX2* production may occur in earlier stages of erythroid development, or there are post-transcriptional processes yet to be explored. In contrast, *β*–thalassemia major patients (n = 8) showed high levels of *PRDX2* when compared to *β*–thalassemia intermedia patients; however, these were inadequate to work together with the reduced *PRDX1* protein level, as mentioned before.

### 6.4. TRX1

Thioredoxin 1 (*TRX1*), located at 9q31.3, acts as an antioxidant as it involves oxidative stress and inflammation, and it is used to recharge *PRDX2* after it reduces hydrogen peroxide [68]. *TRX1* was significantly elevated in *β*–thalassemia intermedia (n = 15) patients compared to healthy individuals (n = 16), indicating that an increase in this protein has an anti-apoptotic function and protects cells against induced cell death by oxidative stress in *β*–thalassemia. *TRX1* and *PRDX1* work synergistically to reduce erythrocyte apoptotic events in *β*–thalassemia intermedia, consequently prolonging their lifespan in the bloodstream [65].

### 6.5. SOD1

The superoxide dismutase 1 (*SOD1*) gene at 21q22.11 encodes an essential enzyme that combats free radicals in erythrocytes. An up-regulation in gene and protein expression of *SOD1* in *β*–thalassemia intermedia patients (n = 15) was observed, compared to healthy individuals (n = 16). This could result from compensatory mechanisms in response to the high levels of ROS observed in *β*–thalassemia intermedia [65].

## 7. Possible Targets for Gene Therapy

The only curative treatment for transfusion-dependent *β*–thalassemia is allogeneic hematopoietic stem cell transplantation. Current advances in technology, such as CRISPR/Cas9, will allow the exploration of possible targets for gene therapy.

### SOX6

A member of the SOX family/group D, *SOX6* is a critical transcription factor responsible for γ– to β–globin switching by suppressing γ-globin gene expression. Disruption of the *SOX6* binding site impairs its binding to the γ-globin promoter, leading to the continuous activation of the γ-globin gene. By utilizing CRISPR/Cas9 technology, Shariati et al. [69] reactivated γ-globin expression by mutating the *SOX6* binding site, and observed an increase in the γ-globin mRNA level up to 2.1-fold in treated K562 cells, when compared with untreated cells. This finding supports the use of CRISPR-mediated *SOX6* disruption as an alternative to reduce the clinical burden of *β*–thalassemia. Down-regulation of *SOX6* expression by lentiviral RNAi in K652 cells, as well as erythroblasts derived from normal donors and *β*–thalassemia major mononuclear cells (MNC), induced γ-globin production in all three cell types without impairing erythroblast maturation, suggesting that *SOX6* could be a target to ameliorate *β*–thalassemia via increased γ-globin levels [70].

**Figure 4:**
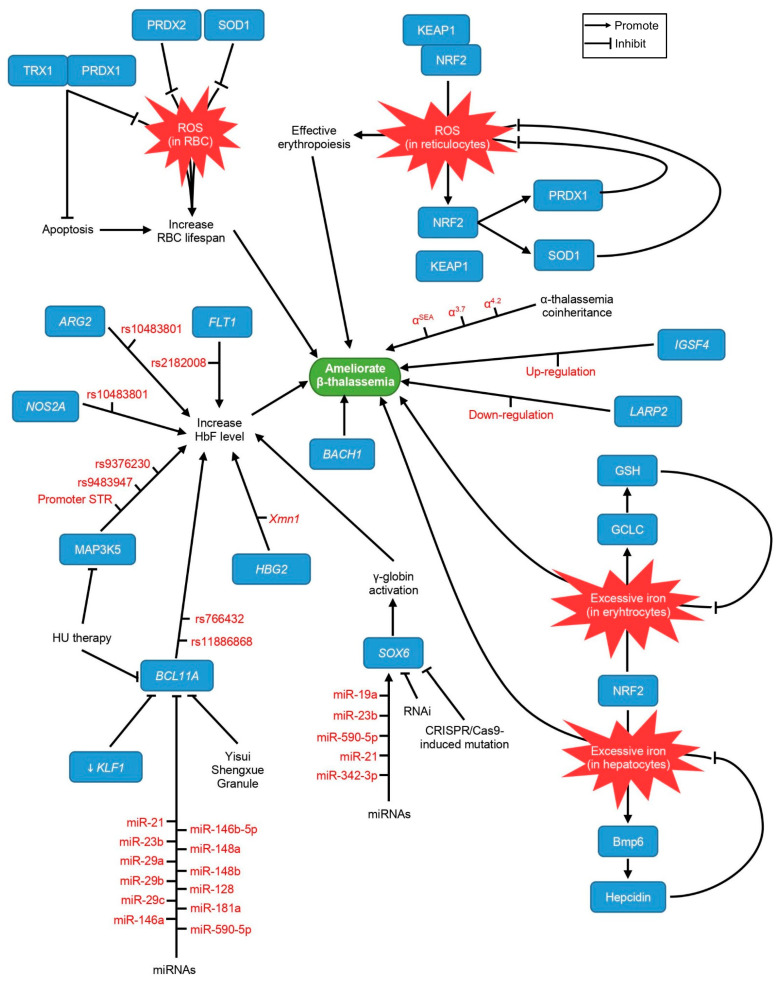
The integration of epigenetic modifiers in ameliorating *β*–thalassemia.

Microarray data demonstrated that five miRNAs might target the *SOX6* gene, namely *miR-19a*, *miR-23b*, *miR-590-5p*, *miR-21*, and *miR-342-3p*, as up-regulation was only found in CD34^+^-derived erythroid cells of HPFH-2 (n = 2), not in δ*β*–thalassemia (n = 2) and healthy controls (n = 3). This outcome suggests that *SOX6* expression is modulated by these miRNAs, thereby influencing HbF levels [35]. In another study, zinc-finger nuclease (ZFN) was used to reactivate γ-globin expression in K652 cells through integrase without lentivirus, which induces mutations in the binding domain of *SOX6*. Upon ZFN treatment, a six-fold increase in γ-globin mRNA levels was demonstrated, compared to untreated cells, signifying that the integrase without lentivirus-mediated ZFN approach could be developed as a therapeutic approach to treat *β*–thalassemia patients [71]. The in vitro response of *SOX6* to HU treatment was investigated in hematopoietic erythroid progenitors derived from the peripheral blood of homozygous IVSII-1G>A *β*–thalassemia patients (HU responders, n = 10, and HU non-responders, n = 10) and healthy individuals (n = 10). *SOX6* expression was down-regulated in the control group (*p* = 0.014). At the same time, no significant changes were observed in the responder (*p* = 0.402) or non-responder (*p* = 0.757) groups after HU treatment, suggesting that in vitro prediction of HU response is not feasible, since each individual has intracellular factors that differ [36]. Figure 4 depicts the influence of these epigenetic modifiers to ameliorate *β*–thalassemia.

## 8. Conclusions

The complexity of the genetic background underlying *β*–thalassemia makes predicting disease outcomes difficult. *β*–thalassemia patients should be asymptomatic. However, some exhibit *β*–thalassemia intermedia phenotypes; meanwhile, those with genetically determined *β*–thalassemia major may clinically present as *β*–thalassemia intermedia. Genetic modifiers rather than β–globin genotypes best explain this remarkable clinical diversity of *β*–thalassemia. The most explored *β*–thalassemia genetic modifiers include methylation status, which down-regulates β–globin synthesis, gene polymorphisms, which elevate γ–globin production, and coinheritance of α–thalassemia, which ameliorates the severity of *β*–thalassemia. Perhaps, in the future, additional genetic modifiers can be explored to understand the clinical diversity of *β*–thalassemia fully, and identify therapeutic approaches to treat this disease effectively.

## Figures and Tables

**Table 1:** Summary of the epigenetic modifiers involved in *β*–thalassemia.

Epigenetic Modifiers Involved	Study Type	Findings	References
**DNA methylation**			
**β–globin cluster**	Experimental study	Hypomethylation in the CpG sites around the LCR HS4-HS3, γ– and β–globin promoter	[15]
**Histone Modification**			
**H3K27**	Experimental study	Thalidomide treatment markedly reduced the H3K27 methylation	[16]
**γ–globin**	Experimental study	Vorinostat induced γ–globin production and simultaneously suppressed α–globin expression	[17]
**MS-275**	Experimental study	MS-275 analogues (MD48) highly induced HbF production	[18]
**ASH1L**	Experimental study	ASH1L expression increased, thus ASH1L binding on the β– and α–globin promoters was also increased	[19]
**IOX1**	Experimental study	IOX1 down-regulated α– and α–like globin expression without affecting β–like globin expression	[20]
**SIRT1**	Experimental study	SIRT1 knockdown decreased, and its overexpression increased the γ–globin gene	[21]
**Other epigenetic modifiers**			
***IGSF4***	Cohort study	*IGSF4* promoter was fully and partially methylated in *β*–thalassemia major and HbE/*β*–thalassemia, respectively	[23]
	Case-control study	High methylation of *IGSF4* in *β*–thalassemia patients	[22]
***LARP2***	Cohort study	Partial methylation of *LARP2* in HbE/*β*–thalassemia and *β*–thalassemia major patients	[24]
	Cohort study	Up-regulation of *LARP2* expression in *β*–thalassemia major	[26]
	Cohort study	Hypermethylation of *LARP2* promoter region	[25]
**Factors involved in the transcription control of the HBB locus**			
***BCL11A***	Experimental study	Down-regulation of *BCL11A* via lentiviral RNA interference (RNAi) reactivated HbF expression	[27]
	Experimental study	Double knockdowns of *BCL11A* and DNMT1 enhanced 90% expression of γ–globin	[30]
	Experimental study	Erythroblasts from bone-marrow had significant expression of *BCL11A* compared to fetal erythroblasts	[31]
	Cohort study	rs11886868 and rs766432 increase HbF level	[33,34]
	Cohort study	Up-regulation of 12 microRNAs targeting *BCL11A* gene, namely miR-21, miR-23b, miR-29a, miR-29b, miR-29c, miR-146a, miR-146b-5p, miR-148a, miR-148b, miR-128, miR-181a, and miR-590-5p may explain the down-regulation of *BCL11A*	[35]
	Experimental study	Hydroxyurea treatment significantly decreased the *BCL11A* expression	[36]
	Cohort study	A complex Chinese medicine, yisui shengxue granule, was demonstrated to down-regulate the *BCL11A* gene expression	[37]
***HBS1L-MYB***	Experimental study	Double knockdowns of MYB and DNMT1 significantly induced ε–globin	[30]
	Cohort study	rs9399137 had no significant effect in modifying HbF level or clinical appearance in both *β*–thalassemia and HbE/*β*–thalassemia	[32]
	Cohort study	rs9399137 frequencies were high in homozygous HbE subjects with high HbF levels	[33]
	Cohort study	rs9376092 was significantly associated with the HbE/β^0^-thalassemia severity	[34]
***KLF1***	Experimental study	KLF1 controls the globin gene switching by directly influence *BCL11A* level and γ–globin/β–globin expression ratios	[13]
	Cohort study	*KLF1* mutation significantly associated with high HbF levels	[40]
	Cohort study	11 *KLF1* mutations were observed in high HbF hemoglobinopathies patients but the mutations were not functionally defective *KLF1* mutations	[41]
	Cohort study	*KLF1* mutations were significantly higher in the patients from the endemic thalassemia region than in the non-endemic thalassemia region	[42]
	Cohort study	KLF1 mutation (NM_006563.4:c.968C>T) was suggested to ameliorate severe *β*–thalassemia genotype, especially homozygous IVS1-110	[39]
	Cohort study	KLF1 and *BCL11A* were inversely correlated with γ–globin gene expression in patients with KLF1 gene mutations	[43]
	Cohort study	KLF1 gene variations were not significantly related to borderline HbA2 *β*–thalassemia carriers	[44]
	Experimental study	The amount of KLF1 expression is weakly positively correlated with *BCL11A* mRNA	[45]
	Experimental study	KLF1 knockdown decreases *BCL11A* expression and elevates HbF levels	[46]
***GATA1***	Experimental study	*GATA1* was suggested to favor binding to the hypomethylated sites in fetal erythroblasts	[31]
	Experimental study	No significant footprint was observed in adult erythroid cells and lymphocytes when the γA promoter is methylated	[48]
	Experimental study	Yisui shengxue granule decreased *GATA1* and GATA2 expressions	[49]
***FLT1***	Cohort study	*FLT1* gene SNP (rs2182008 (G>A)) was strongly associated with the elevation of HbF levels	[50]
***BACH1***	Cohort study	*BACH1*, where it was significantly correlated with age, α–, β– and γ–globin gene expression levels and heme oxygenase-1 protein	[51]

## Data Availability

Not applicable.
